# Increasing access to evidence‐based treatment for child anxiety problems: online parent‐led CBT for children identified via schools

**DOI:** 10.1111/camh.12612

**Published:** 2022-12-13

**Authors:** Iheoma Green, Tessa Reardon, Roberta Button, Victoria Williamson, Gemma Halliday, Claire Hill, Michael Larkin, Falko F. Sniehotta, Obioha C. Ukoumunne, Tamsin Ford, Susan H. Spence, Paul Stallard, Cathy Creswell

**Affiliations:** ^1^ Departments of Experimental Psychology and Psychiatry University of Oxford Oxford UK; ^2^ Oxford Health NHS Foundation Trust Oxford UK; ^3^ School of Psychology and Clinical Language Sciences University of Reading Reading UK; ^4^ Department of Psychology, Institute of Health and Neurodevelopment Aston University Birmingham UK; ^5^ Department of Public Health, Social and Preventive Medicine, Medical Faculty Mannheim Heidelberg University Mannheim Germany; ^6^ NIHR Policy Research Unit Behavioural Science Newcastle University Newcastle upon Tyne UK; ^7^ NIHR ARC South West Peninsula (PenARC) University of Exeter Exeter UK; ^8^ Department of Psychiatry University of Cambridge Cambridge UK; ^9^ Australian Institute for Suicide Research and Prevention, and School of Applied Psychology Griffith University Brisbane Queensland Australia; ^10^ Department for Health University of Bath Bath UK

**Keywords:** Child, anxiety, digital, parents, intervention, CBT

## Abstract

**Background:**

Anxiety problems are extremely common and have an early age of onset. We previously found, in a study in England, that fewer than 3% of children with an anxiety disorder identified in the community had accessed an evidence‐based treatment (Cognitive Behavioural Therapy; CBT). Key ways to increase access to CBT for primary school‐aged children with anxiety problems include (a) proactive identification through screening in schools, (b) supporting parents and (c) the provision of brief, accessible interventions (and capitalising on technology to do this).

**Method:**

We provided a brief, therapist guided treatment called Online Support and Intervention (OSI) to parents/carers of children identified, through school‐based screening, as likely to have anxiety problems. Fifty out of 131 children from 17 Year 4 classes in schools in England screened positive for ‘possible anxiety problems’ and 42 (84%) of these (and 7 who did not) took up the offer of OSI. We applied quantitative and qualitative approaches to assess children's outcomes and families' experiences of this approach.

**Results:**

Inbuilt outcome monitoring indicated session on session improvements throughout the course of treatment, with substantial changes across measures by the final module (e.g. Child Outcome Rating Scale *d* = 0.84; Goal Based Outcomes *d* = 1.52). Parent engagement and satisfaction was high as indicated by quantitative and qualitative assessments, and intervention usage.

**Conclusions:**

We provide promising preliminary evidence for the use of OSI as an early intervention for children identified as having anxiety problems through school‐based screening.

**Key Practitioner Message**

- Anxiety problems often start in childhood and are common. Cognitive Behavioural Therapy (CBT) is an effective treatment, but few children access it.
- An online version of therapist‐supported, parent‐led CBT for child anxiety problems is acceptable, engaging and associated with positive outcomes when delivered to the parents of children identified as having potential anxiety problems through school‐based screening.
- These preliminary findings suggest that OSI may be a valuable tool for increasing access to CBT for children with anxiety problems. Further systematic evaluation is required.

## Introduction

Anxiety disorders often have an early age of onset, with many common anxiety disorders having a median age of onset below 13 years (Solmi et al., 2022). They are also the most prevalent mental health disorders across the lifespan with a worldwide point prevalence between 4.7% and 9.1% among children and young people (Polanczyk, Salum, Sugaya, Caye, & Rohde, 2015). Furthermore, childhood anxiety disorders can have negative impacts on education, social and health functioning (Asselmann, Wittchen, Lieb, & Beesdo‐Baum, 2018). If left unmanaged they can continue into adulthood and can increase the risk of developing other mental health problems (Kendall, Safford, Flannery‐Schroeder, & Webb, 2004). The early onset, high prevalence, combined with the known burden caused by anxiety problems highlight the need for effective, early intervention in childhood.

Effective treatments for childhood anxiety disorders exist (specifically Cognitive Behavioural Therapy, CBT; James, Reardon, Soler, James, & Creswell, 2020), but recent research in England indicated that only 38% of 7–11 year old children with a diagnosable anxiety disorder had accessed any professional support, and only 2% had received CBT (Reardon, Harvey, & Creswell, 2020). Guided parent‐delivered CBT (GPD‐CBT), in which parents or carers (from here on ‘parents’) are supported by a therapist to implement CBT strategies in their child's day to day life, is a low‐intensity intervention that has shown good outcomes for reducing child anxiety disorders within clinical settings. For example, in a randomised controlled trial, 50% of children had recovered from their primary diagnosis (compared to 25% of those on waitlist) after just 5 h of therapist contact, which increased to 76% by the 6‐month follow‐up (Thirlwall et al., 2013).

To date, GPD‐CBT has generally been delivered in face‐to‐face and/or telephone formats. However, we recently developed a digital version (OSI: Online Support and Intervention for child anxiety) through a process of user‐centred‐design with children, parents and clinicians (Hill, Reardon, Taylor, & Creswell, 2022). OSI comprises a parent website, case management system for clinicians and mobile game application for children. It was developed in response to parents' requests for improved access to CBT through engaging, self‐study online content that parents could access at times that suit them. A preliminary evaluation of OSI with children aged 7–12 years in a clinical setting indicated that OSI is feasible, acceptable to families and appears to be associated with positive outcomes within routine clinical practice (Hill, Chessell, Percy, & Creswell, 2022). However, limiting delivery to clinical settings inevitably introduces barriers to access to psychological interventions for children and families. Indeed, known barriers to accessing professional support include difficulties identifying anxiety problems, perceived negative consequences of help‐seeking for the child and/or family and lack of parental knowledge of the help‐seeking processes (e.g., not knowing who to ask for help; Reardon, Harvey, Young, O'Brien, & Creswell, 2018). One potential way to overcome these barriers is to offer support to families of children identified as having likely anxiety problems through routine screening in school settings.

Schools have a central role in the lives of many children. High attendance rates and contact hours and close relationships between school staff and pupils and their families make schools a suitable place to identify and support many children with mental health problems (Soneson et al., 2020). ‘Universal screening’ of children and young people's mental health is increasingly advocated as a means to identify youth who may have unmet mental health needs (Husabo et al., 2020), with the requirement that appropriate access to support should follow these procedures (Humphrey & Wigelsworth, 2016). We have previously successfully codesigned a set of procedures for the identification and treatment of childhood anxiety problems through primary schools in England (Williamson et al., 2021), in which universal screening is followed by feedback to parents and the offer of online GPD‐CBT for child anxiety problems (OSI). The present paper reports the outcomes from an initial evaluation of the use of OSI following screening for anxiety problems in a primary school setting, including evaluation of children's outcomes and families' experiences and engagement with the digital intervention.

## Method

### Design

This study is an uncontrolled case series using a repeated measures design to evaluate child outcomes from OSI, following screening for child anxiety problems in a primary school setting. Qualitative interviews were conducted to explore parents' experiences of OSI. Data were collated from two consecutive studies conducted to test the development and then the feasibility of the approach. Minor differences in procedures across the two studies are highlighted below. The study designs were preregistered (Study 1: Williamson et al., 2021; Study 2: Reardon et al., 2022) and the analytic plan for this case series was prespecified (https://osf.io/basq3/).

### Participants

**Figure 1:**
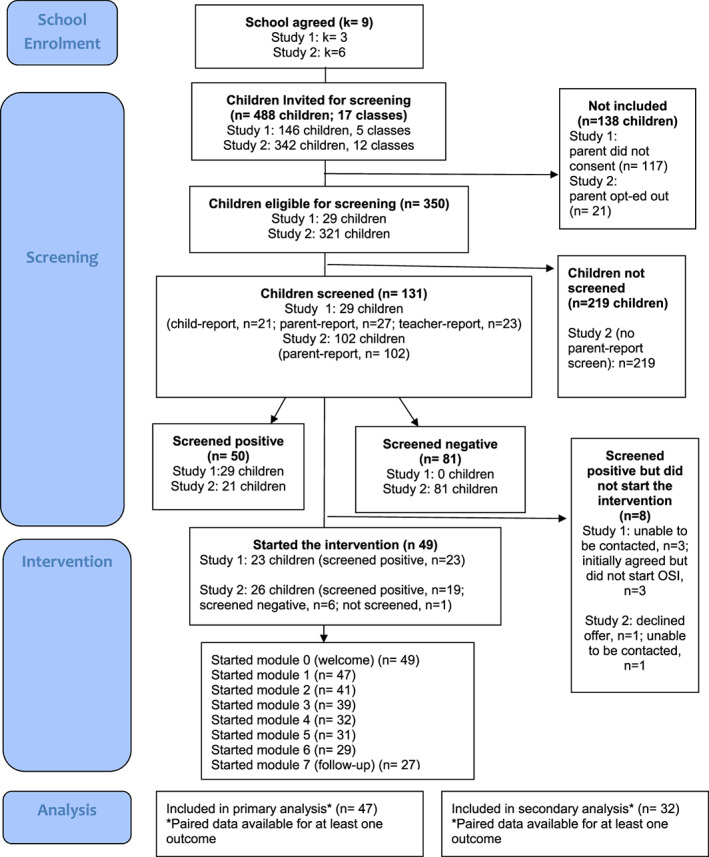
Recruitment and retention flowchart

Participants were children in Year 4 (8–9 years), their parents and class teachers in nine mainstream primary schools in England. Participants were recruited in two phases between March and June 2020 (study 1) and November 2020 and May 2021 (study 2). Participation at each stage is detailed in Figure 1. Of the 488 children in 17 participating classes, screening questionnaires were completed for 131 children, and 50 children screened positive for anxiety problems. Parents of 42 (84%) children who screened positive for anxiety problems took up the offer of support and started OSI. Of the eight families who did not start OSI: one declined the offer as they had alternative support in place; four could not be contacted; and three failed to start OSI following initial acceptance of the offer [time pressure (*n* = 1); unknown (*n* = 2)]. Although screening measures can improve identification, they are rarely 100% accurate so we also let parents of children who did not screen positive know that they could also access the intervention if they wished. Parents of a further 8 children who did not screen positive for anxiety problems requested support through OSI (6 of these screened negative and 2 did not complete the screening). Seven of these families started OSI. In total, 49 parents started OSI and 47 provided paired data (see below) and were included in primary analyses (follow‐up analyses without those that did not screen positive are provided in Tables S1 and S2). Participant characteristics are provided in Table 1.

**Table 1:** Sample characteristics

	Study 1	Study 2	Total
	*N* = 21	*N* = 26	*N* = 47
Child age, mean (*SD*)	8.76 (0.44)	8.69 (0.47)	8.72 (0.45)
Child gender, female, *n* (%)	13 (62)	16 (62)	29 (62)
Child ethnicity			
White British, *n* (%)	13 (62)	24 (92)	37 (79)
Any other White background, *n* (%)	4 (19)	1 (4)	5 (11)
Other ethnic background, *n* (%)	2 (10)	1 (4)	3 (6)
Not stated, *n* (%)	2 (10)	0 (0)	2 (4)
Parent relationship to child			
Mother, *n* (%)	19 (91)	23 (89)	42 (89)
Father/step‐father, *n* (%)	2 (10)	3 (12)	5 (11)
Parent education			
School completion, *n* (%)	1 (5)	3 (12)	4 (9)
Further education, *n* (%)	2 (10)	10 (39)	12 (26)
Higher/postgraduate education, *n* (%)	18 (86)	13 (50)	31 (66)
Parent type of housing			
Private rented, *n* (%)	7 (33)	2 (8)	9 (19)
Council housing/housing association, *n* (%)	0 (0)	1 (4)	1 (2)
Mortgage/fully owned, *n* (%)	12 (57)	23 (89)	35 (75)
Other, *n* (%)	2 (10)		2 (4)

All parents who initiated the intervention were invited to participate in interviews about their experience of the intervention. Characteristics of the 14 parents that took part in qualitative interviews are provided in Table S3.

### Procedures

School headteachers were first asked to provide written agreement for their school to participate in the study. Study information was then distributed on paper and/or online to all children, parents and class teachers in participating classes. In Study 1 parents were required to provide informed written opt‐in consent prior to screening. Because of concerns about families missing out on the opportunity to participate and access support, in Study 2 we used an initial opt‐out recruitment procedure and parents were given the opportunity to opt their child out of screening. Parents, children and their class teachers were then invited to complete questionnaires about the child's anxiety online or on paper. Children provided written assent to participate in the study prior to completing child‐report questionnaires. Parents provided written consent for themselves and their child to participate in the study prior to completing parent‐report questionnaires. Screening outcomes (positive/ negative) were determined from responses to these anxiety questionnaires (see below). All parents were sent a feedback letter outlining the screening results. Those who screened positive for anxiety problems were contacted by a Children's Wellbeing Practitioner (CWP; graduate therapists trained to deliver brief psychological interventions) to offer the online intervention. Because the screening measures are unlikely to detect all children that might benefit from the intervention, parents of children who screened negative and (in Study 2 where an opt‐out initial recruitment method was used) parents who had not completed the parent‐report screening questionnaire were invited to contact the study team if they wished to discuss the online intervention further. If not provided at an earlier stage, informed parent consent was obtained prior to starting OSI. Parents completed outcome questionnaire measures via OSI.

### Intervention

Following screening, parents of eligible children were offered OSI: an online GPD‐CBT intervention for child anxiety problems (Hill, Reardon, et al., 2022; https://osiresearch.org.uk/osi/). OSI is accessible by mobile, tablet, or computer. It comprises eight online modules accessed sequentially, which cover core content of face‐to‐face GPD‐CBT approaches (see Table S4) across six modules bookended by a welcome module that introduces parents to OSI (module 0) and a 4‐week follow‐up (module 7). Each online module takes approximately 20–30 min to complete and is supported by a brief telephone session with a CWP. The modules consist of simple text, audio versions of text, videos and animation, interactive activities and inbuilt routine outcome measures. Parents are also offered access to an optional mobile game app for their child, which is designed to help motivate the child to engage in the treatment strategies. At the start of each online module, parents are required to complete routine outcome measures; other interactive elements (e.g., module questions) are encouraged but optional. CWPs can view parent responses to measures and activities via an accompanying clinician website. Support calls with a CWP accompany each module and are scheduled once a week for 7 weeks with a final call 4‐weeks later; each call takes about 20 min (i.e., approximately 2 hr and 40 min of therapist guidance in total). Parents are encouraged to practice the strategies and skills learnt in the modules ahead of their call. During the calls CWPs help parents to personalise the content and problem solve as required. The two CWPs that delivered OSI were trained in GPD‐CBT (as part of their professional training) and OSI specifically, through written manuals and one to one guidance and supervision. With parental consent, telephone sessions were audio‐recorded for supervision to maintain good clinical practice, and CWPs received regular supervision from clinical psychologists with expertise in treating childhood anxiety disorders.

## Measures

### Screening measures

Screening in Study 1 and Study 2 differed as measurement practices evolved as described in Appendix S1 (and in https://osf.io/ue2cz). In Study 1 children were considered to screen positive for anxiety problems if they scored above the established cut‐off score on the SCAS‐8 on the basis of at least one reporter (child, parent, or teacher) *and* indicated at least ‘a little’ impact on the basis of at least one reporter. In Study 2 we identified children on the basis of a brief parent‐report measure of distress and interference caused by anxiety.

### Intervention outcome measures

Parent‐report outcome and satisfaction measures are built into the online intervention (OSI) and were selected to adhere to the Children and Young People's Improving Access to Psychological Therapies dataset (Wolpert, Curtis‐Tyler, & Edbrooke‐Childs, 2016) with the addition of a measure of life interference caused by child anxiety as this has been found to relate well to diagnostic outcomes (Evans, Thirlwall, Cooper, & Creswell, 2017) and to be valued by young people and parents (Creswell et al., 2021; Krause et al., 2021). Full details are provided in Appendix S2. The prespecified primary outcome of interest was the Child Outcome Rating Scale (parent‐report CORS; Miller, Duncan, Brown, Sparks, & Claud, 2003).

### Engagement with OSI

We used the following to assess engagement with OSI: (a) completion rates for modules, and optional questions and quizzes that parents can choose to complete throughout modules, (b) accuracy of the optional quiz question responses and (c) usage data routinely collected within the online programme, including the number of times each module page was viewed and time spent on each module page. The free‐standing nature of the children's game app meant that we were unable to record usage for the accompanying game.

### Experience of OSI

Qualitative interviews followed an indicative topic guide, which invited parents to share their experiences of the screening and intervention procedures, including their experiences of being offered OSI, of engaging with the treatment modules, and the perceived impact of OSI on their child as well as any unexpected secondary impacts, such as an impact on their overall family functioning or parental confidence. Interviews were conducted on a 1:1 basis. The interviewer had access to supervision, as a forum for discussing the process of data collection, and for exploring any challenges, which arose during that process. Participants were offered the opportunity to take part either via telephone or video conference call (e.g. MS Teams). Due to COVID‐19 restrictions, interviews were not conducted in person. Interviews were audio‐recorded with participant consent and transcribed verbatim.

Interviews were transcribed in full, omitting personally identifying information. Nvivo 12 software was used to facilitate data analysis and organisation. Data were analysed using Template Analysis (King, 2012). Researchers first became familiar with the data by rereading transcripts several times then a template of initial codes was created guided by the interview schedule questions and relevant empirical literature. Transcripts were then analysed in a ‘top down’ fashion following the provisional structure of the templates. Themes relevant to the study research question were identified in the coded data set through analysis of patterns found between codes and among coded segments as well as through code use frequencies. Each theme was discussed and developed through team discussion.

## Results

Our data analytic approach is provided in Appendix S3.

### Session by session clinical outcome data

**Figure 2:**
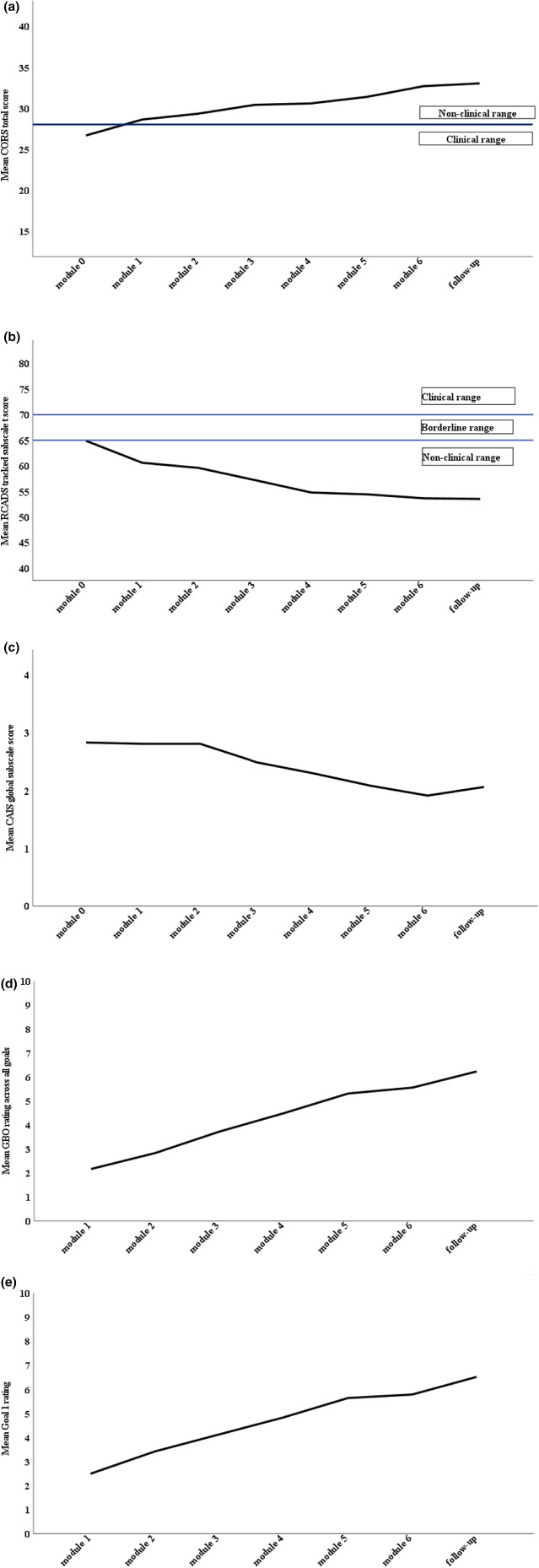
Session by session clinical outcomes. (a). Session by session Child Outcome Rating Scale (CORS) mean total scores (*N* = 47). (b) Session by session Revised Children's Anxiety Scale (RCADS) mean ‘tracked subscale’ *t* score (*N* = 47). *Note:* ‘Tracked subscale’ refers to the RCADS subscale that best reflected the child's main anxiety problem. This was administered at the start of every module. (c) Session by session Child Anxiety Impact Scale (CAIS) mean global subscale score (*N* = 47). (d) Session by session Goal Based Outcome (GBO) mean score across all goals (*N* = 41). (e) Session by session Goal Based Outcome mean first goal score (*N* = 41)

Figure 2 displays the mean session by session scores for the primary (CORS) and secondary clinical (RCADS‐tracked subscale *t* score, CAIS‐ global subscale, GBO across all goals, GBO first goal) outcomes. As can be seen in the figures, for all measures mean scores showed session on session improvement; where cut‐offs are available (CORS, RCADS) mean scores were within the nonclinical range by Module 1 and continued to move further into this range as treatment progressed. A consistent pattern was also seen in the secondary analyses (Figure S1).

### Module 0 to module 6 and module 7 (follow‐up) change in primary and secondary outcomes

As displayed in Table 2, primary and secondary clinical outcomes all showed medium‐large (on basis of conventions; Cohen, 1988) positive effects from Module 0 to Module 6 (*d* = 0.76–1.34) and to Module 7 (*d* = 0.79–1.52), with the exception of CAIS‐P‐global subscale, which showed somewhat smaller positive effects (*d* = 0.43 and 0.35, respectively). Secondary analyses revealed a consistent pattern (Table S5).

**Table 2:** Primary and secondary clinical outcomes

	Module 0^b^	Module 6	Module 7 (Follow‐up)	Module 0 versus Module 6 Cohen's *d*	Module 0 versus Module 7 Cohen's *d*
	(Preintervention)	(Postintervention)			
	Mean (*SD*), *N*	Mean (*SD*), *N*	Mean (*SD*), *N*		
Primary outcome					
CORS total score	26.50 (7.21), *N* = 47	32.32 (7.54), *N* = 47	32.66 (7.49), *N* = 47	0.79	0.84
Secondary outcomes					
RCADS‐P *t* score^a^	63.72 (13.59), *N* = 29	53.21 (13.36), *N* = 29	53.00 (11.35), *N* = 29	0.78	0.86
RCADS‐P‐‘tracked subscale’ *t* score	64.89 (16.18), *N* = 47	53.66 (13.32), *N* = 47	53.53 (12.43), *N* = 47	0.76	0.79
CAIS‐P total score^a^	16.62 (9.68), *N* = 29	7.90 (10.28), *N* = 29	8.52 (8.83), *N* = 29	0.87	0.89
CAIS‐P global subscale score	2.83 (2.44), *N* = 47	1.91 (1.83), *N* = 47	2.06 (1.89), *N* = 47	0.43	0.35
GBO mean score across all goals	2.16 (1.88), *N* = 41^b^	5.55 (3.03), *N* = 41	6.23 (3.25), *N* = 41	1.34	1.52
GBO first goal score	2.49 (2.82), *N* = 41^b^	5.78 (3.35), *N* = 41	6.51 (3.52), *N* = 41	1.06	1.26

‘Tracked subscale’ refers to the RCADS subscale that best reflected the child's main anxiety problem. This was administered at the start of every module.

CORS, Child Outcome Rating Scale; RCADS‐P, Revised Children's Anxiety and Depression anxiety and depression scale; RCADS‐P‐tracked subscale, Revised Children's Anxiety and Depression tracked subscale; CAIS‐P, Child Anxiety Impact Scale; GBO, Goal Based Outcomes.

^a^ Scale only used at Module 0, Module 6 and Follow‐up so it was not possible to replace missing values.

^b^ Preintervention GBO is assessed at Module 1.

At Module 6, 23% (*n* = 11) showed reliable improvement in overall functioning post‐treatment, and this increased to 32% (*n* = 15) at Module 7 (Table 3). Among those children who scored below the cut‐off (<28) on the CORS preintervention, 35% (*n* = 9) and 54% (*n* = 14) were categorised as reliably improved postintervention and at follow‐up, respectively (Table 3). A similar pattern is shown in the secondary analyses (Table S6), with 31% (*n* = 10) and 44% (*n* = 14) reliably improved at Module 6 and Module 7, respectively, and 47% (*n* = 9) and 74% (*n* = 14) among the subset who scored below the cut‐off at Module 0.

**Table 3:** Reliable change in overall functioning (Child Outing Rating Scale)

	Module 6	Module 7
	(Postintervention)	(Follow‐up)
Total sample (*N* = 47)		
Reliable improvement, *n* (%)	11 (23)	15 (32)
No reliable change, *n* (%)	36 (77)	31 (66)
Reliable deterioration, *n* (%)	0 (0)	1 (2)
Below cut‐off (<28) at Module 0 (*N* = 26)		
Reliable improvement, *n* (%)	9 (35)	14 (54)
No reliable change, *n* (%)	17 (65)	12 (46)
Reliable deterioration, *n* (%)	0 (0)	0 (0)

### Treatment satisfaction

**Figure 3:**
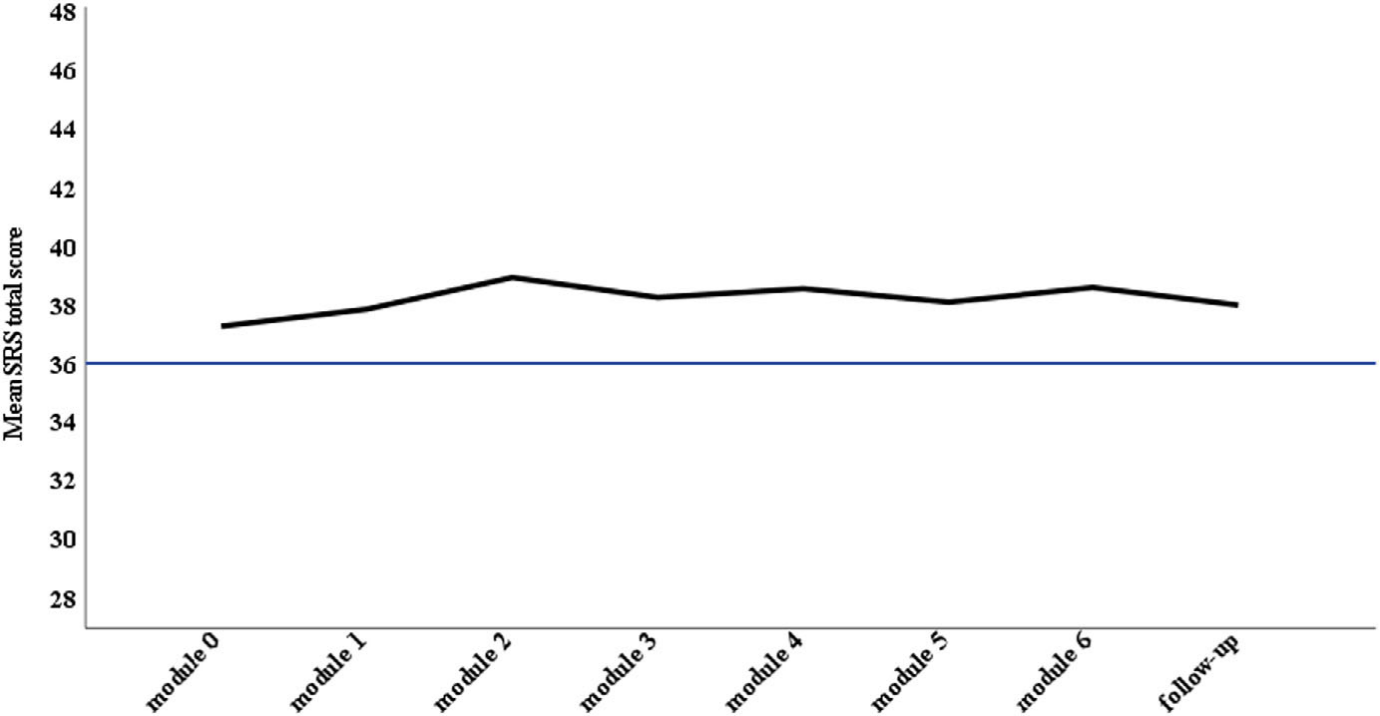
Session by Session Rating Scale (SRS) mean total scores. *Note*. Module 0 (Welcome): *N* = 34; Module 1: *N* = 37; Module 2: *N* = 38; Module 3: *N* = 32; Module 4: *N* = 31: Module 5: *N* = 29; Module 6: *N* = 27; Module 7 (Follow‐up): *N* = 6

Figure 3 displays the session by session SRS ratings. Across all modules, >80% of sessions were rated above the established cut‐off for a good therapeutic relationship (see Table S7). As displayed in Table S8, module feedback responses were positive, with 70%–100% of respondents providing a response of ‘agree’ or ‘strongly agree’ for each item.

### Treatment engagement

Module completion rates indicated a good level of engagement, with 32 of 49 parents who started OSI (65%) completing all of the key intervention components (Modules 0–4), and 27 (55%) completing all modules from 0 to Module 7. Table S9 shows that optional quizzes had high completion rates throughout (median percentage of quiz questions answered, 100% for each module) and quiz question responses indicate a high level of understanding among parents (median percentage correct 100% for each module). The median percentage of optional questions answered ranged from 75% to 90% for early modules (Modules 1–4) where key treatment content is provided, to 50% to 67% for later modules (Modules 5–6). The median number of module pages viewed and median time spent viewing module pages varied across modules, reflecting variation in the amount of content and number of pages across modules (see Table S9). For each module, the median total number of pages viewed (from 12 to 36 pages viewed per module) was higher than the number of pages in the module, indicating parents tended to view pages multiple times, with the median total time spent viewing each module page ranging from 34 min (Module 3, *facing fears*) to 9 min (Module 0, *welcome module*).

### Treatment experience

Three overarching themes were developed to reflect participants' views as described below. Anonymised quotes for each theme are provided in Table S10.

- OSI fits within modern lifestyles

Parents universally reflected that receiving support via an online platform could overcome barriers to treatment, such as mental health‐related stigma, but also make treatment more readily accessible. Parents described being able to easily access OSI when they had time, as well as revisiting the modules as needed. Online delivery was reported by parents to help overcome structural barriers to care, including perceived difficulty taking time off work for an appointment and travelling time.

- OSI can help with both children's anxiety and parent's confidence

A number of parents reported that since engaging with OSI, their confidence in managing their child's anxiety had substantially increased (as well as for supporting other children in the family). The addition of tailored 1:1 support from the CWP was described as reassuring and an effective way to troubleshoot aspects of the modules that were experienced as less straightforward, although some felt that it may take longer to build a relationship with a therapist without face‐to‐face interaction. Joining OSI as part of a research study, with the knowledge that other parents must also be taking part, was also described as destigmatising.

- There can be challenges in using OSI (environment and skills)

Despite the convenience of OSI being online, some parents encountered technical difficulties – such as losing their place – when using the platform. Other parents described how weekly OSI modules could be difficult to fit into their busy schedules and noted that some parents may need more time.

## Discussion

This extended case series provides promising evidence for OSI, an online GPD‐CBT intervention, with children who were identified as having likely anxiety problems through school‐based screening. Built in routine outcome monitoring within OSI ensured high levels of data completion, which demonstrated session on session improvements across all measures, which continued to the follow‐up assessment, 1 month after the core treatment ended. Unsurprisingly, given that this study aimed to evaluate early intervention, some effect sizes from the start to the end of treatment were somewhat smaller than in a previous evaluation of OSI that was conducted within a clinical setting (e.g., CORS pre to follow‐up here *d* = 0.84 compared to 0.96 in Hill, Chessell, et al. (2022)). However, the improvements seen were nonetheless in keeping with those found in trials of far more intensive interventions for child anxiety disorders (e.g. James et al., 2020). Furthermore, as also found by Hill, Chessell, et al. (2022), improvements were particularly large on the Goal Based Outcome measure, which is encouraging given that such idiographic outcome measures may be more sensitive to change than standardised outcome measures (Edbrooke‐Childs, Jacob, Law, Deighton, & Wolpert, 2015). In addition, despite the relatively low baseline scores among this early intervention sample, rates of reliable change and reliable deterioration compare favourably to those found in a recent meta‐analysis of outcomes in routine clinical practice in specialist mental health services (Bear, Edbrooke‐Childs, Norton, Krause, & Wolpert, 2020) although notably that study included children and adolescents whereas our study only included preadolescent children.

Indicators of parent engagement and satisfaction with OSI were extremely positive. Despite this not being a help‐seeking population, the majority of parents who were offered OSI took it up (84%) and almost two‐thirds of parents who started the intervention completed the core content (65%). Parents rated a strong therapeutic alliance with the CWP throughout, the time spent on each module adhered to the amount of content provided (with evidence of parents returning to the content on multiple occasions), and there were high levels of completion of optional module materials. Overall qualitative feedback on OSI was positive. The online platform was seen as more convenient and less stigmatising than traditional face‐to‐face appointments in a clinic. Parents welcomed access to tailored support from the CWPs via weekly telephone calls and felt that these calls personalised the experience of treatment. Consistent with Spencer, Topham, and King (2020) parents also felt that OSI improved their confidence in managing and responding to their child's anxiety symptoms. Nonetheless, as has been found for other online interventions (Hall & Bierman, 2015) some parents encountered technical difficulties or difficulties incorporating weekly OSI sessions into their daily life. These experiences provide useful information for future minor adaptations to OSI that may help the small number of families who experience these difficulties.

Together the findings indicate that OSI may be a valuable tool to provide efficient, engaging, effective support to parents of children with emerging anxiety problems identified within school settings. Indeed, strengths of the approach include the delivery of online support directly to families via schools thus relieving families of multiple barriers that can prevent access to support through specialist services (Reardon et al., 2017). However, several limitations of the study should be acknowledged. First, there was no control group and little is known about the likely change over time in our measures among this targeted group without intervention. Our study was intended to be descriptive (and as such we have avoided inferential statistics) and, to evaluate effectiveness, a randomised controlled trial comparing this approach to usual school practice is required. Furthermore, although we identified participants through school‐based screening, a relatively small proportion (27%) of parents of children in participating classes completed screening measures (Husabo et al., 2020). This is good reason to be cautious as, if it is the case that parents who were especially motivated or had particular resources were more likely to engage, there is a risk that only providing support through this means may increase inequalities in access to services. However, it is also important to note that, nonetheless, a relatively high number, approximately 10%, of the children in participating classes ‘screened positive’, which may suggest that parents who felt that they and/or their children would benefit were more likely to take part. It is also difficult to know how the rates of uptake would differ outside the context of a research study in which there are multiple stages of consent and a heavy burden of measures (outside of OSI) and other administration. Furthermore, this study took place during the COVID‐19 pandemic when schools were closed for several months so direct contact with children, parents and school staff was not always possible. Further evaluation of the implementation of a ‘screening +intervention’ pathway in routine practice (i.e. outside of a formal research study) would be beneficial to establish the extent of parental participation without these constraints and to evaluate whether the approach does successfully increase access to psychological therapies. We focused specifically on children in Year 4 following feedback from schools that this would work well. In addition, overall, the participating sample were less diverse than would be expected of children in England in terms of the number of children from minority ethnic backgrounds (21% vs. 33.69% for England; Gov.uk, 2021) and were relatively affluent as measured on the basis of home ownership (75% vs. 61% among 35–44 year olds in England; Gov.uk, 2020). The participating parents were also predominantly mothers (89%). Further evaluation with more diverse participant groups are clearly required.

In conclusion, this evaluation provides promising preliminary evidence that an online parent‐led CBT intervention (OSI) can be used to provide efficient, engaging, early support for parents of children with anxiety problems. Further systematic evaluation is now required to establish the effectiveness of this school‐based screening and online intervention approach through randomised controlled trials with longer term follow‐ups.

## Author contributions

All authors contributed to study design. I.G., R.B., C.H., G.H. led on intervention delivery and quantitative data collection. I.G. and T.R. led on quantitative data analysis. V.W. and M.L. led on qualitative data collection and analysis. I.G., T.R., V.W. and C.C. led on drafting the manuscript. All other authors reviewed and contributed to the manuscript. C.C. has full access to all the data in the study and takes responsibility for the integrity of the data in the study and the accuracy of the data analysis. The authors would like to thank participating families and schools, all members of the iCATS management group, including our parent representatives, the iCATS research team and our PPI advisors for their invaluable contributions. The authors are also very grateful to Katie Fletcher for administrative support. The authors have declared that they have no competing or potential conflicts of interest.

## Ethical statement

The studies were approved by the University of Oxford Medical Sciences Interdivisional Research Ethics Committee (Study 1: R64620/RE001; Study 2: R71772/RE001).

## Supporting information


**Appendix S1.** Screening procedures.
**Appendix S2.** Built in outcome and satisfaction measures.
**Appendix S3.** Data analysis.
**Figure S1.** Session by session clinical outcomes (secondary analyses).
**Table S1.** Primary and secondary clinical outcomes (among children who screened positive for anxiety problems at baseline) (*N* = 40).
**Table S2.** Reliable change in overall functioning (Child Outing Rating Scale) (among children who screen positive for anxiety problems at baseline).
**Table S3.** Qualitative interview participant characteristics.
**Table S4.** OSI treatment content.
**Table S5.** Primary and secondary clinical outcomes (secondary analysis).
**Table S6.** Reliable change in overall functioning (Child Outing Rating Scale) (secondary analysis).
**Table S7.** Session Rating Scale ratings for each module.
**Table S8.** Module feedback ratings for each module: mean ratings and frequency of ‘strongly agree’ and ‘agree’ responses.
**Table S9.** Treatment engagement: completion of optional questions and quizzes and usage data.
**Table S10.** Treatment experience: Themes following template analysis and illustrative quotes.
