# Supplementary material for: Increasing access to evidence‐based treatment for child anxiety problems: online parent‐led CBT for children identified via schools
Source: Child Adolesc Ment Health. 2022 Dec 13;28(1):42–51. doi: 10.1111/camh.12612 (PMC10108299; doi:10.1111/camh.12612)
Supplement: Supplementary file 1 — Appendix S1. Screening procedures. Appendix S2. Built in outcome and satisfaction measures. Appendix S3. Data analysis. Figure S1. Session by session clinical outcomes (secondary analyses). Table S1. Primary and secondary clinical outcomes (among children who screened positive for anxiety problems at baseline) (N = 40). Table S2. Reliable change in overall functioning (Child Outing Rating Scale) (among children who screen positive for anxiety problems at baseline). Table S3. Qualitative interview participant characteristics. Table S4. OSI treatment content. Table S5. Primary and secondary clinical outcomes (secondary analysis). Table S6. Reliable change in overall functioning (Child Outing Rating Scale) (secondary analysis). Table S7. Session Rating Scale ratings for each module. Table S8. Module feedback ratings for each module: mean ratings and frequency of ‘strongly agree’ and ‘agree’ responses. Table S9. Treatment engagement: completion of optional questions and quizzes and usage data. Table S10. Treatment experience: Themes following template analysis and illustrative quotes. [file CAMH-28-42-s001.docx]

**Supporting Information**

**Table S1.**

Primary and secondary clinical outcomes (among children who screened positive for anxiety problems at baseline) (N=40)

|  | Module 0*  (Pre-intervention)  Mean (SD), N | Module 6  (Post-intervention)  Mean (SD), N | Module 7 (Follow-up)    Mean (SD), N | Module 0 versus Module 6 Cohen’s *d* | Module 0 versus Module 7 Cohen’s *d* |  |
| --- | --- | --- | --- | --- | --- | --- |
| Primary outcome | | | | | | |
| CORS  total score | 26.07 (7.29), N=40 | 31.91 (7.76), N=40 | 32.17 (7.80), N=40 | 0.78 | 0.81 |  |
| Secondary outcomes | | | | | | |
| RCADS-P  *t*-score** | 64.08 (13.55), N=26 | 54.19 (12.62), N=26 | 53.85 (10.57), N=26 | 0.76 | 0.85 |  |
| RCADS-P-“tracked subscale”  *t*-score | 66.13 (16.01), N=40 | 54.98 (12.26), N=40 | 54.95 (11.95), N=40 | 0.79 | 0.80 |  |
| CAIS-P  total score** | 15.69 (9.44), N=26 | 8.15 (10.60), N=26 | 8.73 (8.47), N=26 | 0.75 | 0.78 |  |
| CAIS-P  global subscale score | 2.80 (2.37), N=40 | 2.03 (1.85), N=40 | 2.20 (1.91), N=40 | 0.37 | 0.28 |  |
| GBO  mean score across all goals | *2.15 (1.69), N=35 | 5.40 (2.90), N=35 | 6.09 (3.11), N=35 | 1.41 | 1.64 |  |
| GBO  first goal score | *2.60 (2.80), N=35 | 5.77 (3.16), N=35 | 6.34 (3.48), N=35 | 1.06 | 1.19 |  |

*Pre-intervention GBO is assessed at Module 1. **Scale only used at Module 0, Module 6 and Follow-up so it was not possible to replace missing values

*Note:* “Tracked subscale” refers to the RCADS subscale that best reflected the child’s main anxiety problem. This was administered at the start of every module.

CORS=Child Outcome Rating Scale. RCADS-P=Revised Children’s Anxiety and Depression anxiety and depression scale. RCADS-P-tracked subscale= Revised Children’s Anxiety and Depression tracked subscale. CAIS-P=Child Anxiety Impact Scale. GBO-Goal Based Outcomes

**Table S2.**

Reliable change in overall functioning (Child Outing Rating Scale) (among children who screen positive for anxiety problems at baseline)

|  | Module 6  (Post-intervention) | Module 7  (Follow-up) |
| --- | --- | --- |
| Total sample (N=40) | | |
| Reliable improvement, n (%) | 10 (25) | 13 (33) |
| No reliable change, n (%) | 30 (75) | 26 (65) |
| Reliable deterioration, n (%) | 0 (0) | 1 (3) |
| Below cut-off (<28) at Module 0 (N=23) | | |
| Reliable improvement, n (%) | 8 (35) | 12 (52) |
| No reliable change, n(%) | 15 (66\|) | 11 (48) |
| Reliable deterioration, n (%) | 0 (0) | 0 (0) |

**Table S3.**

Qualitative interview participant characteristics

|  | Child’s screening outcome | Child gender | Parent gender | Parent age | Parent ethnicity | Parent highest level of education | Type of housing | Did the parent start OSI? | Did the parent complete all OSI modules? |
| --- | --- | --- | --- | --- | --- | --- | --- | --- | --- |
| 1 | Screen positive | Male | Female | 40-44 | White British | Higher education/ postgraduate | Rented | Yes | Yes |
| 2 | Screen positive | Female | Male | 45-49 | White British | Higher education/ postgraduate | Rented | Yes | Yes |
| 3 | Screen positive | Female | Female | 40-44 | White British | Higher education/ postgraduate | Mortgage | Yes | No  (completed Modules 0 to 3) |
| 4 | Screen positive | Male | Female | * | * | * | * | Yes | No  (completed Module 0) |
| 5 | Screen positive | Female | Female | 40-44 | Other White Background | Higher education/  postgraduate | Rented | Yes | Yes |
| 6 | Screen positive | Female | Female | 40-44 | Ethnicity not stated | Higher education/ postgraduate | Mortgage | Yes | Yes |
| 7 | Screen positive | Female | Female | 40-44 | Other White Background | Higher education/ postgraduate | Rented | Yes | Yes |
| 8 | Screen negative | Female | Female | 40-44 | White British | Higher education/  postgraduate | Mortgage | Yes | No  (completed Modules 0 to 2) |
| 9 | Screen negative | Male | Female | 40-44 | White British | Further education | Mortgage | Yes | Yes |
| 10 | Screen negative | Male | Male | 45-49 | White British | Higher education/  postgraduate | Mortgage | Yes | No  (completed Modules 0 to 1) |
| 11 | Screen positive | Male | Female | 35-39 | White British | * | Rented | Yes | Yes |
| 12 | Screen positive | Female | Female | 40-44 | White British | * | Mortgage | Yes | Yes |
| 13 | Screen positive | Female | Female | 40-44 | White British | Higher education/  postgraduate | Mortgage | Yes | Yes |
| 14 | Screen positive | Female | Female | 40-44 | White British | Higher education/  postgraduate | Mortgage | Yes | Yes |

*Missing data

**Table S4.**

OSI treatment content

| **Module** | **Topics covered** |
| --- | --- |
| Module 0: Welcome | Overview of treatment and how to use OSI |
| Module 1: Get Ready | Psychoeducation on the development and maintenance of child anxiety. Identification of treatment goals.  Optional interactive activities: maintenance of child anxiety worksheet, parent’s take-home messages, end-of-module quiz. |
| Module 2: Have-A-Go Thinking | Identifying children’s anxious thoughts/how to talk to children about their fears and worries. Exploring thoughts and alternatives to reassurance giving.  Optional interactive activities: exploring thoughts worksheet, parents’ take-home messages, end-of-module quiz. |
| Module 3: Facing Fears | Rationale for exposure, and setting up exposure plan to test fears (including the development and implementation of a step-by-step plan to help children to gradually test their fears and worries).  Optional interactive activities: step-by-step plan worksheet, parent’s take-home messages, end-of-module quiz. |
| Module 4: Becoming Independent & Controlling Worries | Encouraging children to become more independent and management of excessive worry (including worry boxes and worry time).  Optional interactive activities: increasing independence worksheet, parent’s take-home messages, end-of-module quiz. |
| Module 5: Problem Solving | Rationale for, and overview of, problem solving  Optional interactive activities: problem solving worksheet, parent’s take-home messages, end-of-module quiz. |
| Module 6: Keep it Going | Rationale for relapse prevention. Review of treatment techniques that have been helpful. Identification of current and future goals.  Optional interactive activities: current and future goals worksheet, things that have been helpful for my child worksheet, parent’s take-home messages. |
| Module 7: Follow Up | ROMS only - no new content presented |

**Appendix S1.**

Screening procedures

Study 1

*Brief Spence Children’s Anxiety Scale* (Child-, Parent-, and Teacher-report; SCAS-C-8, SCAS-P-8, SCAS-T-8; (Reardon et al., 2018)). Each version of the brief questionnaire includes eight items derived from the full-length SCAS (Spence, 1998) to assess a child’s anxiety symptoms. Items are rated on a 4-point scale ranging from 0 (never) to 3 (always) and total scores reflect the sum of responses to the eight items. In line with previous research, cut-off scores to indicate presence of elevated child anxiety symptoms were 7.5 (SCAS-P), 6.5 (SCAS-C) and 4.5 (SCAS-T) (Reardon et al., 2018). Reports from a community sample show that the SCAS-C/P/T-8 have good internal consistency (α  = .80 - .84), agreement between reporters, and convergent/divergent validity (Reardon et al., 2018).

An *impact* scale was devised to accompany the SCAS-8 because child anxiety impact measures (e.g., parent-report version of the Child Anxiety Impact Scale) have been found to be good predictors of the presence/absence of common anxiety disorders (Evans et al., 2017). The scale asked whether fears, worries, or anxiety cause problems for the child; how long any difficulties with anxiety have been present; how distressing they are for the child; how much the difficulties stop the child from doing activities; and about the perceived need for help/support. Each item is rated on a four-point scale, from 0 to 3. Response options for each item were ‘Not all at’ (0), ‘Only a little’ (1), ‘Quite a lot (2)’, ‘A great deal’ (3), with the exception of the item related to duration of difficulties which was rated as ‘Less than a month’ (0), ‘1-5 months’ (1), ‘6-12 months’ (2), ‘Over a year’ (3).

Children were considered to screen positive for anxiety problems if they scored above the established cut-off score on the SCAS-8 on the basis of at least one reporter *and* scored 1 or more on the ‘impact’ scale on the basis of at least one reporter.

Study 2

*Brief child anxiety screen*. A 2-item parent-report questionnaire assessed the extent to which a child’s fears, worries or anxiety cause distress (‘Do fears, worries or anxiety upset or distress your child?’) and interfere with family life (‘Do your child’s fears, worries or anxiety make things difficult for your family as a whole?’). Items were rated on a 4-point scale, from 0 (‘No, not at all’) to 3 (‘Yes, a great deal’), and responses summed to produce a total score. Children who scored ≥ 3 out of 6 screened positive for anxiety problems as we have previously found that this cut-off identifies children with anxiety disorders from within a community population with 76% sensitivity and 80% specificity (<https://osf.io/ue2cz>).

**References**

Evans, R., Thirlwall, K., Cooper, P., & Creswell, C. (2017). Using symptom and interference questionnaires to identify recovery among children with anxiety disorders. *Psychological Assessment*, 29, 835-843.

Reardon, T., Spence, S. H., Hesse, J., Shakir, A., & Creswell, C. (2018). Identifying children with anxiety disorders using brief versions of the Spence Children’s Anxiety Scale for children, parents, and teachers. *Psychological Assessment*, *30*, 1342-1355.

Spence, S. H. (1998). A measure of anxiety symptoms among children. *Behaviour Research and Therapy*, 36(5), 545-566.

**Appendix S2.**

Built in outcome and satisfaction measures

*Prespecified primary outcome measure*

The *Child Outcome Rating Scale* (parent-report CORS; Miller et al., 2003) is a four-item scale that assesses areas of life functioning that change in response to therapeutic intervention: symptom distress, interpersonal wellbeing, social life, and overall wellbeing. Each dimension is displayed as a visual analogue scale ranging from 0 to 10 (10 = better functioning). The maximum total score is 40 and the parent-report cut-off for good functioning is ≥28. The CORS has good concurrent validity with other outcome measures; and has high internal consistency, α  = .93 and test-retest reliability (Duncan et al., 2006). As prespecified (<https://osf.io/basq3/>), as the families in this study were from a community population and may have had sub-clinical problems with anxiety, the CORS was selected as the primary outcome measure as it monitors change in life functioning, rather than clinical impairment. Parents completed the CORS at the start of each OSI module (Module 0 to Module 7 (follow-up)).

*Secondary clinical outcome measures*

*The Revised Children’s Anxiety and Depression Scale* (parent version; RCADS-P; (Chorpita et al., 2000)) is a 47-item questionnaire that measures symptoms of anxiety and depression. This questionnaire is sub-divided into six scales for generalised anxiety, social phobia, separation anxiety, panic disorder, obsessive compulsive disorder and depression. Each item is scored on a 4-point Likert scale: 0 (never), 1 (sometimes), 2 (often), 3 (always) and parents rate how true each statement is for their child. The RCADS-P shows adequate to excellent internal consistency, α = 0.60–0.96 and good convergent validity in non-clinical samples (Donnelly et al., 2018). Parents completed the full RCADS-P at the start of Module 0, Module 6, and Module 7, and also completed the RCADS-P subscale that best reflected the child’s main difficulty at the start of Modules 1 through to 5. This RCADS-P “tracked subscale” varied across participants, including social phobia (n=18), separation anxiety (n=10), generalised anxiety (n=9), panic disorder (n=6), obsessive compulsive disorder (n=3), depression (n=1). The RCADS-P total anxiety and depression score (RCADS-P) (Module 0, Module 6, Module 7) and the “tracked subscale” score (Module 0-Module 7) were calculated and published norms according to school year group and gender (Chorpita et al., 2005) were used to calculate corresponding *t*-scores.

*The Child Anxiety Impact Scale* (parent version; CAIS-P; (Langley et al., 2004)) is a 25-item parent-report questionnaire that assesses the impact of anxiety on school, social, and home/family activities, and four further items related to global impact. Two additional items from the original scale that are not usually relevant for primary-school aged children (related to dating/having a boyfriend/girlfriend) were not included (as in Evans et al., 2017). Each item is scored on a 4-point Likert scale: 0 (Not at all), 1 (Just a little), 2 (Pretty much), 3 (Very much). The CAIS-P has very good internal consistency, α  = .70 - .85 (Langley et al., 2014) and convergent validity with other measures of child anxiety (Langley et al., 2014). Parents completed the full CAIS-P at the start of Module 0, Module 6, and Module 7, and the four items related to global impact at the start of Modules 1-5. Items related to impact on school, social, home/family activities were summed to provide a total impairment score (CAIS-P-total; potential range 0-75) (Module 0, Module 6, Module 7), and items related to global impact were summed to provide a global subscale score (CAIS-P-global; potential range 0-12; Module 0 to Module 7).

*The Goal Based Outcomes* (GBO; Law & Jacob, 2015) scale is an idiographic outcome measure that is used to track progress during intervention. Up to three goals are set per family from Module 1 and reviewed throughout OSI (from Module 1 to follow-up). Parent ratings were recorded on a scale from 0 to 10, with 0 reflecting no progress towards the goal and 10 meaning the goal has been fully reached. Because parents were not required to set three goals in all cases, we have provided data on both: (i) mean across all provided goals, and (ii) progress against the first goal.

*The Session Rating Scale* (SRS; Duncan et al., 2003) is a four-item visual analogue scale that measures dimensions of therapeutic relationships including: respect and understanding, relevance of goals and topics, client-practitioner fit, and overall alliance. Each dimension is displayed as a visual analogue scale ranging from 0 to 10. The total maximum score is 40, with higher scores indicating higher client ratings of the therapeutic relationship. In line with the established cut-off, a total score of ≥36 was considered an acceptable therapeutic relationship. The measure shows high internal consistency, α = .89 to .95, and good concurrent validity (Campbell & Hemsley, 2009). Parents completed the SRS following each session call (Module 0 to Module 7), before they started the next module.

*Module Feedback Questionnaire* (Hill et al., 2022). The Module Feedback Questionnaire includes 10-items that measures module acceptability. Items are scored on a 5-point Likert scale: 1 (Strong disagree), 2 (Disagree), 3 (Neutral), 4 (Agree), 5 (Strongly agree). Parents completed the Module Feedback Questionnaire at the end of each module (Module 0 to Module 6) before the session call.

**References**

Campbell, A., & Hemsley, S. (2009). Outcome Rating Scale and Session Rating Scale in psychological practice: Clinical utility of ultra‐brief measures. *Clinical Psychologist*, 13, 1-9.

Chorpita, B. F., Yim, L., Moffitt, C., Umemoto, L. A., & Francis, S. E. (2000). Assessment of symptoms of DSM-IV anxiety and depression in children: A revised child anxiety and depression scale. *Behaviour Research and Therapy*, 38, 835-855.

Chorpita, B. F., Moffitt, C. E., & Gray, J. (2005). Psychometric properties of the Revised Child Anxiety and Depression Scale in a clinical sample. *Behaviour Research and Therapy*, 43, 309-322.

Donnelly, A., Fitzgerald, A., Shevlin, M., & Dooley, B. (2019). Investigating the psychometric properties of the revised child anxiety and depression scale (RCADS) in a non-clinical sample of Irish adolescents. *Journal of Mental Health*, 28, 345-356.

Duncan, B. L., Miller, S. D., Sparks, J. A., Claud, D. A., Reynolds, L. R., Brown, J., & Johnson, L. D. (2003). The Session Rating Scale: Preliminary psychometric properties of a “working” alliance measure. *Journal of Brief Therapy*, 3, 3-12.

Duncan , B., Sparks, J. A., Miller, S. D., Bohanske, R. T., & Claud, D. A. (2006). Giving youth a voice: A preliminary study of the reliability and validity of a brief outcome measure for children, adolescents, and caretakers. *Journal of Brief Therapy*, 5, 71-88.

Evans, R., Thirlwall, K., Cooper, P., & Creswell, C. (2017). Using symptom and interference questionnaires to identify recovery among children with anxiety disorders. *Psychological Assessment*, 29, 835-843.

Hill, C., Reardon, T., Taylor, L., & Creswell, C. (2022). Online Support and Intervention for Child Anxiety (OSI): Development and Usability Testing. *JMIR Formative Research*, 6(4), e29846.

Langley, A. K., Bergman, R. L., McCracken, J., & Piacentini, J. C. (2004). Impairment in childhood anxiety disorders: Preliminary examination of the child anxiety impact scale–parent version. *Journal of Child and Adolescent Psychopharmacology*, 14, 105-114.

Langley, A. K., Falk, A., Peris, T., Wiley, J. F., Kendall, P. C., Ginsburg, G., Birmaher, B., March, J., Albano, A. M., & Piacentini, J. (2014). The child anxiety impact scale: examining parent-and child-reported impairment in child anxiety disorders. *Journal of Clinical Child & Adolescent Psychology*, 43, 579-591.

Law, D., & Jacob, J. (2015). Goals and goal based outcomes (GBOs). London: CAMHS Press.

Miller, S. D., Duncan, B., Brown, J., Sparks, J., & Claud, D. (2003). The outcome rating scale: A preliminary study of the reliability, validity, and feasibility of a brief visual analog measure. *Journal of Brief Therapy*, 2, 91-100.

**Appendix S3.**

Data analysis

*Quantitative data analysis*

Data were analysed using IBM SPSS Statistics 27. As prespecified, for clinical outcomes primary analyses of change between Module 0 and Module 6 (end of core content) and change between Module 0 and Module 7 (follow-up), included all participants where there were paired data available for the relevant outcome (i.e., data were available for Module 0 and at least one subsequent module up to and including the target time point). Missing data were replaced with the last available routine outcome measure collected within OSI for that participant. Secondary analyses were also conducted only including participants who completed, at a minimum, the routine outcome measures for the first five online modules (Modules 0-4) as these participants had received the key intervention components. For these secondary analyses, missing data were replaced with the last available time point from Module 5 onwards.

Mean session-by-session scores (from Module 0 to Module 7) were calculated for the primary outcome (CORS total score) and secondary clinical outcomes that were completed on a weekly basis (RCADS-tracked subscale *t*-score, CAIS-P-global subscale score, GBO mean across all goals, GBO first goal score). Mean Module 0, Module 6, and Module 7 scores were calculated for the remaining secondary clinical outcomes (RCADS-P *t*-score, CAIS-P-total score). To determine the magnitude of change following the intervention repeated measures effect sizes (Cohen’s d) were calculated by dividing the difference between means by the average standard deviation (Lakens, 2013). The effect size for the primary (CORS total score) and secondary clinical outcomes (RCADS-P *t*-score; RCADS-tracked subscale *t*-score; CAIS-P-total score; CAIS-global subscale score; GBO-mean across all goals; GBO-first goal score) were calculated from Module 0 to Module 6, and from Module 0 to Module 7. We had not pre-specified that we would calculate Module 0 to Module 6 effect sizes for the secondary clinical outcomes but added these to ensure a consistent approach across all clinical outcomes.

As prespecified, for the primary outcome (CORS total score), we also calculated the reliable change criterion to determine whether change in scores was greater than would be expected from measurement error alone. In line with recent research (Edbrooke-Childs et al., 2018) reliable change for the CORS total score was calculated using the formulae below, using the scale’s internal consistency (Cronbach alpha) as a measure of reliability and the total score standard deviation for our pre-intervention observations: Reliable change criterion = SEdiff x 1. 96, where SEdiff = SD x √2 x √(1 – reliability). We categorised Module 0 to Module 6 change scores, and Module 0 to Module 7 change scores, as: i) reliably improved (improvement >reliable change criterion), ii) reliably deteriorated (deterioration > reliable change criterion), iii) no reliable change (change score does not cross reliable change criterion). As pre-specified, reliable change is reported for the total sample, and for the subsample who scored below the CORS cut-off (<28) pre-intervention.

To assess treatment satisfaction we calculated: i) mean session-by-session SRS ratings, and the number and percentage of participants who scored above the SRS cut-off (≥36) for each session, and ii) mean ratings and frequency of ‘strongly agree’ and ‘agree’ responses for each module feedback questionnaire item. Summary descriptive statistics are also presented for each treatment engagement measure.

**References**

Edbrooke-Childs, J., Wolpert, M., Zamperoni, V., Napoleone, E., & Bear, H. (2018). Evaluation of reliable improvement rates in depression and anxiety at the end of treatment in adolescents. *BJPsych Open*, 4, 250-255.

Lakens, D. (2013). Calculating and reporting effect sizes to facilitate cumulative science: a practical primer for t-tests and ANOVAs. *Frontiers in psychology*, 4, 863. https://doi.org/10.3389/fpsyg.2013.00863

**Figure S1.**

Session by session clinical outcomes (secondary analyses)

1A Session by session Child Outcome Rating Scale (CORS) mean total scores (N=32)


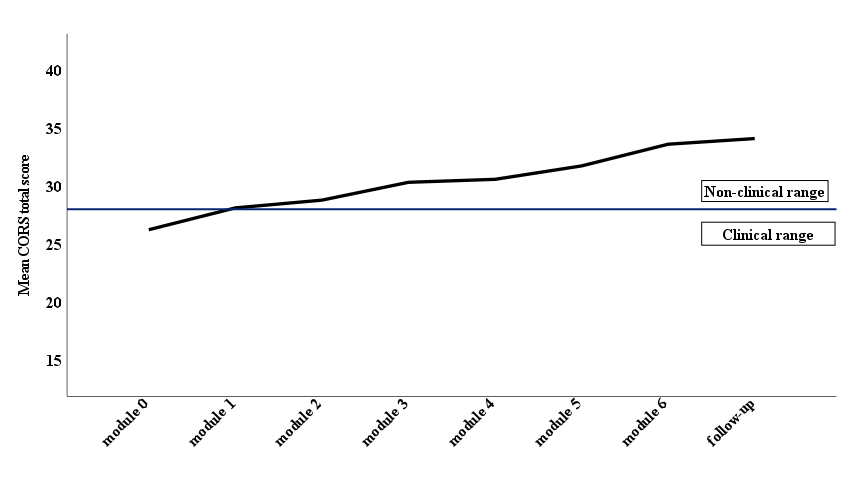


1B Session by session Revised Children’s Anxiety Scale (RCADS) mean “tracked subscale” t scores (secondary analysis) (N=32)


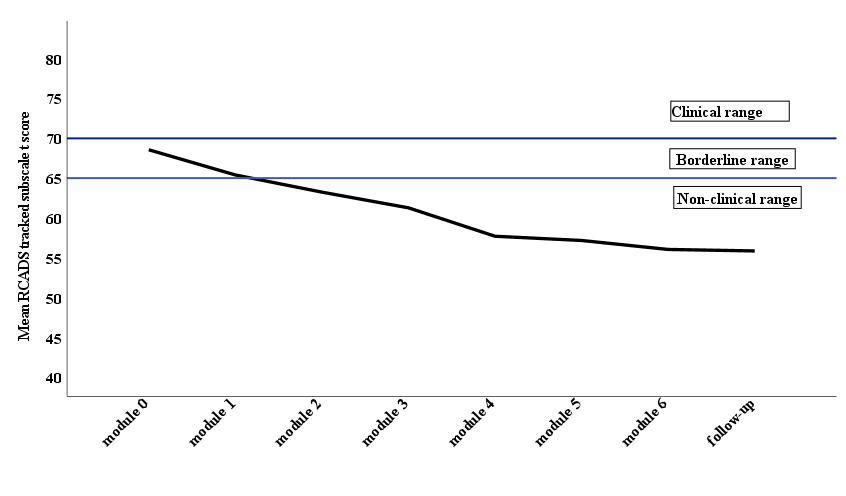


*Note:* “Tracked subscale” refers to the RCADS subscale that best reflected the child’s main anxiety problem. This was administered at the start of every module.

1C Session by session Child Anxiety Impact Scale (CAIS) mean global subscale score (secondary analysis) (N=32)


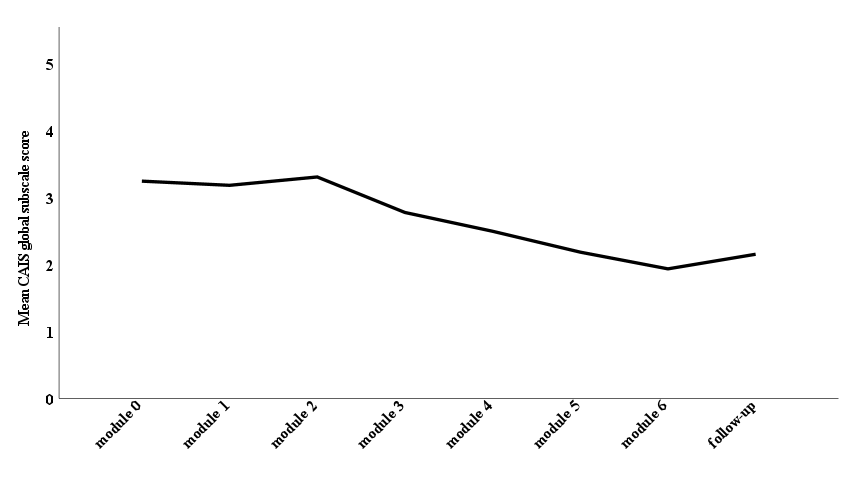


1D Session by session Goal Based Outcome (GBO) mean score across all goals (secondary analysis) (N=32)


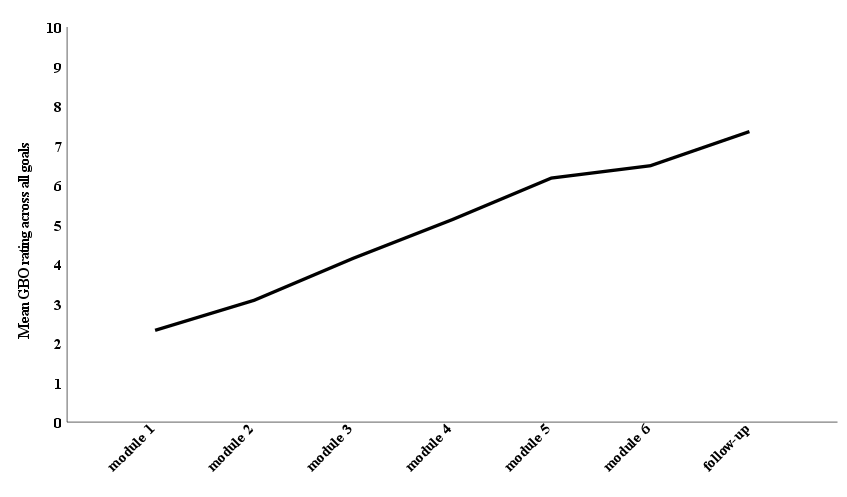


1E Session by session Goal Based Outcome mean first goal score (secondary analysis) (N=32)


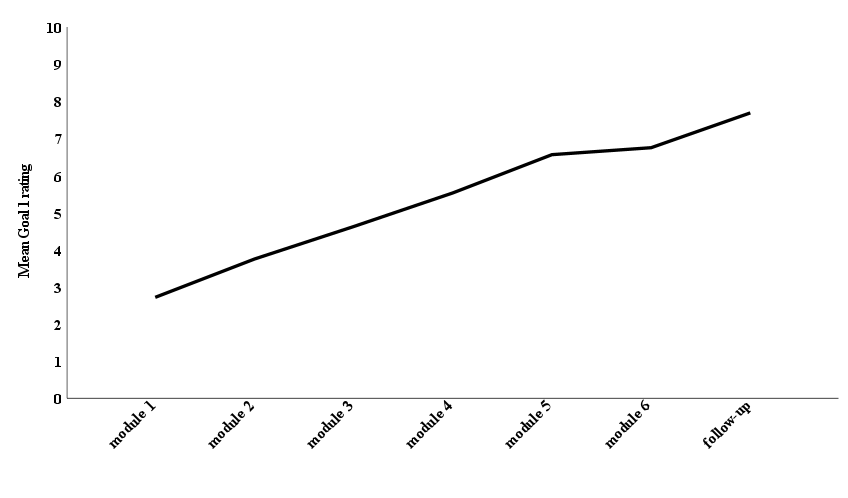


**Table S5.**

Primary and secondary clinical outcomes (secondary analysis)

|  | Module 0*  (Pre-intervention)  Mean (SD), N | Module 6  (Post-intervention)  Mean (SD), N | Module 7  (Follow-up)  Mean (SD), N | Module 0 versus Module 6 Cohen’s *d* | Module 0 versus Module 7 Cohen’s *d* |
| --- | --- | --- | --- | --- | --- |
| **Primary outcome** |  |  |  |  |  |
| CORS  total score | 26.23 (7.15), N=32 | 33.61 (6.78), N=32 | 34.09 (6.59), N=32 | 1.06 | 1.14 |
| **Secondary outcomes** |  |  |  |  |  |
| RCADS-P-tracked subscale  *t*-score | 68.56 (17.45), N=32 | 56.06 (14.33), N=32 | 55.88 (13.14), N=32 | 0.78 | 0.82 |
| CAIS-P  global subscale score | 3.25 (2.49), N=32 | 1.94 (1.78), N=32 | 2.16 (1.87), N=32 | 0.61 | 0.50 |
| GBO  mean score across all goals | 2.32 (1.96), N=32 | 6.49 (2.70), N=32 | 7.35 (2.68), N=32 | 1.77 | 2.15 |
| GBO  first goal score | 2.72 (2.98), N=32 | 6.75 (2.97), N=32 | 7.69 (2.87), N=32 | 1.36 | 1.70 |

*Pre-intervention GBO is assessed at Module 1.

CORS=Child Outcome Rating Scale. RCADS-P-tracked subscale= Revised Children’s Anxiety and Depression tracked subscale. CAIS-P=Child Anxiety Impact Scale. GBO-Goal Based Outcomes

**Table S6.**

Reliable change in overall functioning (Child Outing Rating Scale) (secondary analysis)

|  | Module 6  (Post-intervention) | Module 7  (Follow-up) |
| --- | --- | --- |
| Total sample (N=32) | | |
| Reliable improvement, n (%) | 10 (31) | 14 (44) |
| No reliable change, n(%) | 22 (69) | 17 (53) |
| Reliable deterioration, n (%) | 0 (0) | 1 (3) |
|  | | |
| Below cut-off (<28) pre-intervention (Module 0) (N=19) | | |
| Reliable improvement, n (%) | 9 (47) | 14 (74) |
| No reliable change, n(%) | 10 (53) | 5 (26) |
| Reliable deterioration, n (%) | 0 (0) | 0 (0) |

**Table S7.**

Session Rating Scale ratings for each module

|  | Module 0 | Module 1 | Module 2 | Module 3 | Module 4 | Module 5 | Module 6 | Module 7 (Follow-up) |
| --- | --- | --- | --- | --- | --- | --- | --- | --- |
| Session Rating Scale total score  Mean (SD), N | 37.97 (3.71), N=34 | 38.33 (2.76), N=37 | 38.74 (2.11), N=38 | 39.41 (1.35), N=32 | 39.46  (1.12), N=31 | 39.39 (1.63), N=29 | 39.57 (1.25), N=27 | 38.32 (2.13),  N=6 |
| n (%) ≥ 36, indicating acceptable session | 28 (82.4) | 31 (83.8) | 32 (84.2) | 31 (96.9) | 30 (96.8) | 27 (93.1) | 26 (96.3) | 5 (83.3) |

**Table S8.** Module feedback ratings for each module: mean ratings and frequency of ‘strongly agree’ and ‘agree’ responses

| Item^1^ | Module 0 (N=47)  mean (SD)  n (%) strongly agree/agree | Module 1 (N=41)  mean (SD)  n (%) strongly agree/agree | Module 2 (N=38)  mean (SD)  n (%) strongly agree/agree | Module 3 (N=36)  mean (SD)  n (%) strongly agree/agree | Module 4 (N=29)  mean (SD)  n (%) strongly agree/agree | Module 5 (N=30)  mean (SD)  n (%) strongly agree/agree | Module 6 (N=26)  mean (SD)  n (%) strongly agree/agree |
| --- | --- | --- | --- | --- | --- | --- | --- |
| The module was easy to understand | 4.70 (0.46)  47 (100) | 4.36 (0.58)  40 (95)** | 4.39 (0.55)  37 (97) | 4.36 (0.59)  34 (94) | 4.69 (0.47)  29 (100) | 4.31 (0.76)  26 (90)* | 4.62 (0.70)  25 (96) |
| The module took an appropriate amount of time to complete | 4.47 (0.58)  45 (96) | 4.27 (0.71)  37 (90) | 4.26 (0.60)  35 (92) | 4.06 (0.92)  29 (81) | 4.52 (0.51)  29 (100) | 4.40 (0.62)  28 (93) | 4.58 (0.58)  25 (96) |
| The module was helpful | 3.98 (0.74)  36 (77) | 4.22 (0.73)  36 (88) | 4.38 (0.55)  36 (97)* | 4.25 (0.65)  32 (89) | 4.55 (0.51)  29 (100) | 4.23 (0.63)  27 (90) | 4.35 (0.80)  23 (89) |
| The tone of the material was sensitive for parents seeking help for their child’s anxiety | 4.39 (0.54)  45 (98)* | 4.51 (0.60)  39 (95) | 4.55 (0.56)  37 (97) | 4.50 (0.56)  35 (97) | 4.66 (0.48)  29 (100) | 4.60 (0.50)  30 (100) | 4.65 (0.49)  26 (100) |
| The material was relevant for parents seeking help for their child’s anxiety | 4.17 (0.73)  40 (85) | 4.51 (0.60)  39 (95) | 4.55 (0.50)  38 (100) | 4.42 (0.55)  35 (97) | 4.55 (0.57)  28 (97) | 4.57 (0.50)  30 (100) | 4.62 (0.50)  26 (100) |
| The module was easy to navigate | 4.55 (0.54)  46 (98) | 4.29 (0.84)  35 (85) | 4.39 (0.55)  37 (97) | 4.44 (0.61)  34 (94) | 4.62 (0.49)  29 (100) | 4.38 (0.68)  28 (97)* | 4.65 (0.85)  25 (96) |
| Each screen had the right amount of information | 4.38 (0.61)  44 (94) | 4.49 (0.55)  40 (98) | 4.45 (0.50)  38 (100) | 4.44 (0.65)  35 (97) | 4.66 (0.48)  29 (100) | 4.50 (0.51)  30 (100) | 4.62 (0.57)  25 (96) |
| The module was visually pleasing to me | 4.21 (0.72)  39 (83) | 4.17 (0.67)  35 (85) | 4.32 (0.66)  34 (90) | 4.39 (0.73)  33 (92) | 4.59 (0.57)  28 (97) | 4.40 (0.56)  29 (97) | 4.62 (0.64)  24 (92) |
| It was always clear what to do next in this module | 4.40 (0.58)  45 (96) | 4.17 (0.77)  34 (83) | 4.42 (0.55)  37 (97) | 4.39 (0.60)  34 (94) | 4.64 (0.49)  28 (100)* | 4.20 (0.96)  26 (87) | 4.50 (0.99)  24 (92) |
| I would recommend this module to other parents of anxious children | 4.06 (0.82)  33 (70) | 4.37 (0.66)  37 (90) | 4.47 (0.51)  38 (100) | 4.50 (0.51)  36 (100) | 4.55 (0.51)  29 (100) | 4.47 (0.57)  29 (97) | 4.58 (0.70)  25 (96) |

^1^Rated on a 5-point Likert scale from 1 (strongly disagree) to 5 (strongly agree) . *1 participant missing response. **1 additional response (n=42)

**Table S9.**  Treatment engagement: completion of optional questions and quizzes and usage data

| Module | Number of participants who accessed module* | Number of questions in module | Percentage of questions completed** | | Number of quiz questions in module | Percentage of quiz questions answered** | | Percentage of quiz questions answered correctly*** | | Number of pages in module | Total number of pages viewed ** | | Total time (minutes) spent on module** | |
| --- | --- | --- | --- | --- | --- | --- | --- | --- | --- | --- | --- | --- | --- | --- |
|  |  |  | mean (SD), N | median (IQR), N |  | mean (SD), N | median (IQR), N | mean (SD), N | median (IQR), N |  | mean (SD), N | median (IQR), N | mean (SD), N | median (IQR), N |
| 0 | 47 | 0 | N/A | N/A | 0 | N/A | N/A | N/A | N/A | 11 | 16.02 (7.32), N=47 | 12.0 (10.0),  N=47 | 10.67 (6.36),  N=47 | 9.45 (9.7),  N=47 |
| 1 | 47 | 8 | 81.65 (23.72), N=47 | 87.5 (25.0), N=47 | 5 | 82.98 (37.99), N=47 | 100 (0),  N=47 | 98.97 (4.47), N=39 | 100 (0),  N=39 | 20 | 38.21 (23.16),  N=47 | 32.0 (22.0),  N=47 | 41.47 (32.63),  N=47 | 29.8 (35.8),  N=47 |
| 2 | 41 | 5 | 78.05 (20.40), N=41 | 80.0 (40.0),  N=41 | 7 | 89.20 (26.87) N=41 | 100 (7.1),  N=41 | 92.48 (14.37), N=38 | 100 (14.3),  N=38 | 18 | 31.05 (22.88),  N=41 | 21.0 (18.0),  N=41 | 30.83 (23.51),  N=41 | 25.7 (19.7)  N=41 |
| 3 | 39 | 12 | 70.09 (22.19), N=39 | 75.0 (25.0)  N=39 | 13 | 89.55 (30.69), N=39 | 100 (0),  N=39 | 95.82 (6.28), N=35 | 100 (7.7),  N=35 | 26 | 47.15 (38.36), N=39 | 36.0 (19.0),  N=39 | 121.63 (436.10),  N=39 | 33.64 (38.7)  N=39 |
| 4 | 32 | 10 | 84.38 (18.48), N=32 | 90.0 (20.0)  N=32 | 6 | 90.63 (29.61), N=32 | 100 (0),  N=32 | 98.28 (5.17), N=29 | 100 (0),  N=29 | 19 | 27.88 (21.01), N=32 | 21.5 (12.0),  N=32 | 28.54 (26.79), N=32 | 19.1 (26.6)  N=32 |
| 5 | 31 | 2 | 58.06 (38.94), N=31 | 50.0 (50.0)  N=31 | 6 | 90.86 (26.11), N=31 | 100 (0),  N=31 | 91.95 (17.03), N=29 | 100 (8.3),  N=29 | 18 | 30.16 (32.97), N=31 | 20.0 (13.0),  N=31 | 29.44 (51.33), N=31 | 18.6 (11.8)  N=31 |
| 6 | 29 | 3 | 56.90 (35.78), N=29 | 66.7 (58.3)  N=29 | 0 | N/A | N/A | N/A | N/A | 11 | 17.03 (10.77), N=29 | 14.0 (9.0),  N=29 | 13.04 (11.65), N=29 | 9.8 (10.8)  N=29 |

*Note.* IQR=inter-quartile range (75^th^ percentile – 25% percentile). N/A=not applicable.

*viewed at least one module page **among participants who accessed the module *** among participants who completed at least one quiz question.

**Table S10.**

Treatment Experience: Themes following template analysis and illustrative quotes

| Themes | Illustrative verbatim quotes |
| --- | --- |
| OSI fits within modern lifestyles | *Parent (female): Face-to-face is great but at the same time for a lot of parents it’s that stigma isn’t it around things like this, so actually when they can do it online and really take that information away, I think that’s a really positive thing whereas actually getting people face –to-face, not all will take that. Some will shy away from that and think ‘I don’t want to have someone to tell me that I’m doing something wrong.’*  *Parent (female): Once I got the hang of logging in...I could literally pick [OSI] up and put it down when I had time during the week. Whereas a face –to-face clinic it could involve taking time off work, booking days off or time off, travelling time to get to wherever you are meeting and all that whereas at home I could just if I had ten minutes ...I could do some.* |
| OSI can help with both children’s anxiety and parent’s confidence | *Parent (female): I feel much more confident now definitely without a doubt....With this program ... it’s written in a really positive way ... because you can feel as a parent that somehow you caused [your child’s anxiety].*  *Parent (female): I’ve found it really enlightening and it’s nice to have the encouragement from [the CWP] as well to know that you are not the only one, there are lots of things that you are doing right and there are other ways that you could do things where perhaps you’re not getting the results you want. So it’s been encouraging and really helpful.* |
| There can be challenges in using OSI (environment and skills) | *Parent (Male): I have had some problems locating where I’m at [in OSI] and stuff like that. I’ve been using it on my iPad mainly and I don’t know whether that means there’s a difference in how it looks ...But knowing which of the menu items to click on ... and stuff like that is not necessarily straight forward but... a sort of ‘you are here’ button would be quite handy so you can go back to where you should be. You should be on this page of this module or whatever and that might be useful.*  *Parent (female): One of the suggestions we wanted to make ... would be to consider spacing it out so...you have two weeks to do each module rather than one week because we found that ..., it usually was a few days into the week before we could find time to read the module and then often it took us two or three nights because we just get to it after the kids went to bed. Trying to catch up on work and then we’d be like ‘oh no we’ve got to do the module’ so we’d get on and it would be 11pm at night and we’d get through half of it and then fall asleep. So it would take us several nights to get through it and then by the time we would get through it we didn’t have time to put it into place before our next call. So if we’d had two weeks then we could have spent a week really trying to get through the module and get set up and then spent a week putting it into place and then had the conversation with [the CWP] about what worked and didn’t work. So I just think it would give parents a bit more time to try the things out.* |
